# Gastroesophageal reflux disease is associated with a more severe interstitial lung disease in systemic sclerosis in the EUSTAR cohort

**DOI:** 10.1093/rheumatology/keaf016

**Published:** 2025-01-08

**Authors:** Eliane Roth, Cosimo Bruni, Liubov Petelytska, Mike Oliver Becker, Rucsandra Dobrota, Suzana Jordan, Carina Mihai, Sinziana Muraru, Patricia E Carreira, Jeska De Vries-Bouwstra, Yolanda Braun-Moscovici, Vasiliki Liakouli, Gianluca Moroncini, Christina Bergmann, Luc Mouthon, Christopher P Denton, Maria De Santis, Alberto Cauli, Sabine Adler, Vera Bernardino, Marie-Elise Truchetet, Madelon Vonk, Francesco Del Galdo, Anna-Maria Hoffmann-Vold, Oliver Distler, Muriel Elhai, EUSTAR Collaborators, Serena Guiducci, Ulrich Walker, Florenzo Iannone, Radim Becvar, Otylia Kowal Bielecka, Maurizio Cutolo, Francesco Ciccia, Elise Siegert, Simona Rednic, Yannick Allanore, Panayiotis Vlachoyiannopoulos, Carlomaurizio Montecucco, Murat Inanc, Maria Martin, Beatriz Joven, Cioly Mendez, Srdan Novak, Gábor Kumánovics, Michele Iudici, Przemyslaw Kotyla, Elisabetta Zanatta, Katja Perdan-Pirkmajer, Bernard Coleiro, Silvia Svegliati, Devis Benfaremo, Chiara Paolini, Silvia Agarbati, Dominique Farge Bancel, Paolo Airò, Kristofer Andréasson, Mislav Radic, Alexandra Balbir-Gurman, Andrea Lo Monaco, Nicolas Hunzelmann, Annamaria Iagnocco, Luca Idolazzi, Josko Mitrovic, Voon Ong, Annalyn Nunag, Hanneke Knaapen, Sander van Leuven, Rogier Thurlings, Jelena Colic, Jörg Henes, Vera Ortiz-Santamaria, Johannes Pflugfelder, Dorota Krasowska, Samuel Rubeli, Michaela Köhm, Ivan Foeldvari, Gianluigi Bajocchi, José António Pereira da Silva, Bojana Stamenkovic, Antonio Tonutti, Francesca Motta, Claudia Ickinger, Nimmisha Govind, Lidia P Ananieva, Michael Hughes, Philipp Klemm, Ulf Müller-Ladner, Klaus Søndergaard, Merete Engelhart, Gabriella Szücs, Carlos de la Puente, Øyvind Midtvedt, Torhild Garen, Håvard Fretheim, Mona-Lovise Talaro Ramsli, David Launay, Valeria Riccieri, Andra Balanescu, Ami A Shah, Ana Maria Gheorghiu, Andreas Wirsching, Janina Auth, Alina Ramming, Havvanur Kartalcik, Francesca Ingegnoli, Bertrand Dunogue, Benjamin Chaigne, Vanessa Smith, Francesco Paolo Cantatore, Mette Mogensen, Carlos Alberto von Mühlen, Felix Lauffer, Piotr Wiland, Marie Vanthuyne, Juan Jose Alegre-Sancho, Martin Aringer, Ellen De Langhe, Branimir Ani, Sule Yavuz, Brigitte Granel, Carolina de Souza Müller, Svetlana Agachi, Margarita Pileckyte, Simon Stebbings, Alessandra Vacca, Percival D Sampaio-Barros, Kamal Solanki, Douglas Veale, Esthela Loyo, Walid Ahmed Abdel Atty Mohamed, Jacek Olas, Edoardo Rosato, Figen Yargucu Zhini, Cristina-Mihaela Tanaseanu, Rosario Foti, Codrina Ancuta, Britta Maurer, Marzena Olesinska, Cristiane Kayser, Nihal Fathi, Jorge Juan González Martín, Sophie Blaise, Patricia Senet, Emmanuel Chatelus, Ira Litinsky, Martial Koenig, Sabrina Hoa, Jean-Luc Senécal, Rajvinder Cheema, Begonya Alcacher Pitarch, Lorraine Green, Vishal Kakkar, Stefano Di Donato, Goda Seskute, Lesley Ann Saketkoo, Eduardo Kerzberg, Breno Valdetaro Bianchi, Ivan Castellví, Jasminka Milas-Ahic, Roberta Visevic, Massimiliano Limonta, Doron Rimar, Maura Couto, Camillo Ribi, Antonella Marcoccia, Sarah Kahl, Vivien M Hsu, Thierry Martin, Sergey Moiseev, Lorinda S Chung, Tim Schmeiser, Dominik Majewski, Anna Wojteczek, Julia Martínez-Barrio, Dinesh Khanna, Ana Catarina Rodrigues, Gabriela Riemekasten, Lelita Santos, Yair Levy, Elena Rezus, Daniel Brito De Araujo, Rossella Talotta, Sara Bongiovanni, Marek Brzosko, Hadi Poormoghim, Marta Mamani, Ina Kötter, Giovanna Cuomo, Oscar Massimiliano Epis, Petros Sfikakis, Juliana Markus, Daniel Furst, Ana-Maria Ramazan, Hans Ulrich Scherer, Tom W J Huizinga, Estibaliz Lazaro, Alain Lescoat, Marco Matucci-Cerinic, Julia Spierings, Lidia Rudnicka, Susana Oliveira, Fabiola Atzeni, Masataka Kuwana, Arsene Mekinian, Mickaël Martin, Yoshiya Tanaka, Hidekata Yasuoka, Carmen-Pilar Simeón Aznar, Tatsuya Atsumi, Magda Pârvu, Gonçalo Boleto, Nicoletta Del Papa, Kastriot Kastrati, Jennifer Ben Shimol, Anna Bazela-Ostromecka, Enrico Selvi, Yasushi Kawaguchi, Tomas Soukup, Andrea Nuñez Conde, Marija Geroldinger-Simic, Ignasi Rodríguez-Pintó, Karen Voigt, Torsten Kubacki, Olena Garmish, Marta Mosca, Ulrich Gerth, Marta Dzhus, Tomonori Ishii, Duygu Temiz Karadag, Anastas Batalov, Knarik Ginosyan, Vahan Mukuchyan, Valentina Vardanyan, Armine Haroyan, Tuulikki Sokka-Isler, Len Harty, Mariela Geneva-Popova, Mohammad Naffaa, Cristina Maglio, Cristiana Isabel Sieiro Santos, Okada Masato, Futoshi Iwata, Monique Hinchcliff, Samar Tharwat, Ana Cordeiro, Roberto Giacomelli, Francesco Benvenuti

**Affiliations:** Department of Rheumatology, University Hospital Zurich and University of Zurich, Zurich, Switzerland; Department of Rheumatology, University Hospital Zurich and University of Zurich, Zurich, Switzerland; Department of Rheumatology, University Hospital Zurich and University of Zurich, Zurich, Switzerland; Department of Rheumatology, University Hospital Zurich and University of Zurich, Zurich, Switzerland; Department of Rheumatology, University Hospital Zurich and University of Zurich, Zurich, Switzerland; Department of Rheumatology, University Hospital Zurich and University of Zurich, Zurich, Switzerland; Department of Rheumatology, University Hospital Zurich and University of Zurich, Zurich, Switzerland; Department of Rheumatology, University Hospital Zurich and University of Zurich, Zurich, Switzerland; Rheumatology Department, Hospital Universitario, 12 De Octubre, Madrid, Spain; Department of Rheumatology, Leiden University Medical Center, Leiden, The Netherlands; Rappaport Faculty of Medicine, Rheumatology Institute Rambam Health Care Campus, Technion, Haifa, Israel; Rheumatology Unit, Department of Precision Medicine, University of Campania “Luigi Vanvitelli”, Naples, Italy; Clinica Medica, Department of Internal Medicine, Marche University Hospital, Ancona, Italy; Department Internal Medicine 3, University Hospital Erlangen, Erlangen, Germany; Department of Internal Medicine, Hôpital Cochin, Paris, France; Division of Medicine, Department of Inflammation and Rare Diseases, Centre For Rheumatology Royal Free Hospital and University College London, London, UK; IRCCS Humanitas Research Hospital, Rozzano, Italy; Humanitas University, Pieve Emanuele, Italy; Rheumatology Unit, AOU and University Hospital of Cagliari, Monserrato (CA), Italy; Department of Rheumatology and Immunology, Kantonsspital Aarau, Aarau, Switzerland; Unidade de Doenças Autoimunes—Hospital Curry Cabral, Centro Hospitalar Universitário Lisboa Central, ULS S. José, Centro Clínico Académico de Lisboa, Portugal; Department of Rheumatology, Bordeaux University Hospital, Bordeaux, France; Department of Rheumatology, Radboud University Medical Center, Nijmegen, The Netherlands; Leeds Institute of Rheumatic and Muskuloskeletal Medicine, University of Leeds, Leeds, UK; Department of Rheumatology, University Hospital Zurich and University of Zurich, Zurich, Switzerland; Department of Rheumatology, Oslo University Hospital, Oslo, Norway; Department of Rheumatology, University Hospital Zurich and University of Zurich, Zurich, Switzerland; Department of Rheumatology, University Hospital Zurich and University of Zurich, Zurich, Switzerland

**Keywords:** systemic sclerosis, interstitial lung disease, gastroesophageal reflux disease, progression

## Abstract

**Objectives:**

Gastroesophageal reflux disease (GERD) is frequent in systemic sclerosis (SSc) and could predict progression of interstitial lung disease (ILD). We aimed to analyse (1) the prevalence of GERD among SSc-ILD patients, (2) its association with disease characteristics and (3) predictive factors for ILD progression in SSc-ILD patients with GERD.

**Methods:**

SSc patients from the EUSTAR database with ILD were included. GERD was labelled as present if reflux/dysphagia was reported at the baseline visit or before. Disease characteristics of patients with and without GERD were compared at baseline. ILD progression was defined as relative FVC decline ≥10% or relative FVC decline between 5–9% in association with relative DLCO decline of ≥15% over 12 ± 3 months of follow-up. Prognostic factors for ILD progression, overall survival and progression-free survival in SSc-ILD patients with GERD were tested by multivariable Cox regression.

**Results:**

A total of 5462 SSc-ILD patients were included, 4400 (80.6%) had GERD. Patients with GERD presented more frequently with diffuse cutaneous SSc (OR: 1.44 [1.22–1.69], *P* < 0.001) and more severe lung involvement with lower FVC (85.8 ± 22.1 *vs* 90.2 ± 20.1, *P < *0.001), lower DLCO (60.8 ± 19.7 *vs* 65.3 ± 20.6, *P < *0.001) and worse performance at the 6-min walking test. Female sex (HR: 1.39 [1.07–1.80], *P* = 0.012) and older age (HR: 1.02 [1.01–1.03], *P < *0.001) independently predicted ILD progression in SSc-ILD patients with GERD.

**Conclusion:**

SSc-ILD patients with GERD appear to suffer from a more severe SSc disease. In this population, female sex may be considered a risk factor for ILD progression.

Rheumatology key messages

- Reflux is present in 80% of SSc-ILD patients.
- Patients with SSc-ILD with GERD have from a more severe lung disease.
- In SSc-ILD with GERD, female sex is a risk factor for ILD progression.

## Introduction

Systemic sclerosis (SSc) is a severe autoimmune disease that affects multiple organ systems, including the lungs and gastrointestinal tract. Interstitial lung disease (ILD) is a frequent manifestation of the disease with a prevalence of about 50% of the patients [1]. ILD is the leading cause of morbidity and mortality in SSc, with no significant changes in mortality rate in recent decades [2–4]. In the European Scleroderma Trials & Research Group (EUSTAR) cohort, over 60% of patients with SSc-ILD showed a deterioration in lung function over an average follow-up of 5 years [5]. However, the individual prognosis remains difficult to predict ranging from stable or slowly progressive to rapidly progressive courses [5]. Male sex, older age, African-American ethnicity, diffuse cutaneous SSc, positive anti-topoisomerase I antibodies, low functional vital capacity (FVC) and diffusing capacity for carbon monoxide (DLCO) have been identified as predictive factors for ILD progression [2, 5, 6]. In recent years, some reports have also shown that gastroesophageal reflux disease (GERD) independently predicts ILD progression in SSc [5, 7–9], as well as in idiopathic pulmonary fibrosis [10, 11].

GERD is one of the most common manifestations in SSc, observed in up to 90% of cases [12–15]. It is hypothesized that GERD contributes to ILD through recurrent microaspirations, which might lead to chronic inflammatory reactions and remodeling of the lung structure [10, 11]. Until now, information about characteristics of SSc-ILD patients with GERD has been limited. In a post-hoc analysis of the Scleroderma Lung Study (SLS) II, the reflux score was only associated with dyspnea and cough, but not with other clinical disease characteristics [9]. However, this study included only 142 patients, with a short disease duration and it was a selected population for a clinical trial. Therefore, real-life data on larger cohorts are needed to better define the characteristics of SSc-ILD patients with GERD.

Overall evidence suggests that GERD may contribute to the progression of ILD [5, 15] and PPI may have a protective effect on the progression of lung disease [16]. However, to obtain a larger body of evidence, prospective randomized controlled clinical trials need to be conducted; for example, an intervention trial with PPI to evaluate their effect on SSc-ILD. In order to develop cohort enrichment strategies for progressive ILD patients in such a trial, it is important to identify factors predictive of progression of ILD in this subpopulation.

Using the EUSTAR database, we aimed to better define the phenotype of SSc-ILD patients with GERD in real life and to identify predictive factors for ILD worsening in this population.

## Methods

### Study design

EUSTAR is a large, international, multicentre, prospective registry for SSc patients, representing the largest SSc cohort currently available, with longitudinal follow-up data [3, 5, 17]. For the present approved EUSTAR project CP-142, the EUSTAR cohort database was queried in April 2023 and yielded data for 22860 patients from 237 centres. The structure of the database, the minimal essential data set and the available clinical, demographic and diagnostic parameters have already been described in detail [18]. This study was performed and reported according to the Strengthening the Reporting of Observational studies in Epidemiology (STROBE) statement (Supplementary Data S1, available at *Rheumatology* online) [19]. Each participating centre obtained approval from their local ethics committee and all registered patients granted their written informed consent.

### Patient population and characteristics

Patients fulfilling the 2013 classification criteria for SSc by American College of Rheumatology/European League Against Rheumatism (ACR/EULAR) [20] with ILD diagnosed on high-resolution computed tomography (HRCT) and data available on GERD were included. ILD was defined as ground glass opacities, traction bronchiectasis or reticulation or honeycombing on HRCT regardless of pulmonary function test results [21]. The first visit with ILD on HRCT was set as baseline visit. GERD was labelled as present if reflux/dysphagia was reported at least once before or at the baseline visit [22]. Patients without reflux/dysphagia but use of PPI were excluded from the analysis, as it was difficult to classify them with certainty as GERD with controlled symptoms under therapy or non-GERD.

The following characteristics were extracted from the database: age, sex, ethnicity, smoking status, disease duration defined from first non-Raynaud sign or symptom, cutaneous subset according to LeRoy classification [23], auto-antibody status, C-reactive protein (CRP) levels, digital ulcers and internal organ involvement. The latter included stomach and intestinal symptoms, renal crisis and left heart dysfunction (defined by left ventricular ejection fraction <50% on transthoracic echocardiography).

Parameters from pulmonary function tests (FVC% predicted, DLCO% predicted) were collected. Furthermore, other lung characteristics such as respiratory symptoms (dyspnea according to NYHA classification [24]), oxygen (O2) saturation at rest and 6-min walking distance in metres were inquired.

Recorded treatments at the baseline visit included ILD modifying treatment (defined as an umbrella term for the subcategories of treatments that have demonstrated effects on ILD [25]: cyclophosphamide, mycophenolate mofetil, tocilizumab, rituximab, nintedanib, autologous stem cell transplantation and lung transplantation), corticosteroids and proton pump inhibitors.

For the longitudinal analysis, we included patients with at least two visits 12 ± 3 months apart and with data available on pulmonary function tests (FVC% pred and/or DLCO% pred) to allow the assessment of ILD progression [26, 27]. Patients with pulmonary hypertension on right heart catheterization (RHC) defined by mean pulmonary artery pressure (peroxidase–antiperoxidase) > (20 mmHg [28]) at any time were excluded to avoid bias in the interpretation of DLCO changes.

Progression of ILD was defined as relative FVC%pred decline ≥10% or relative FVC%pred decline between 5–9% in association with relative DLCO%pred decline of ≥15% over 12 ± 3 months follow-up [26, 27]. Progression-free survival was defined as the time from the first visit until progression of ILD and/or death. Overall survival was defined as the time from first visit with ILD until death. All-cause mortality was assessed at last available follow-up.

**Figure 1.**
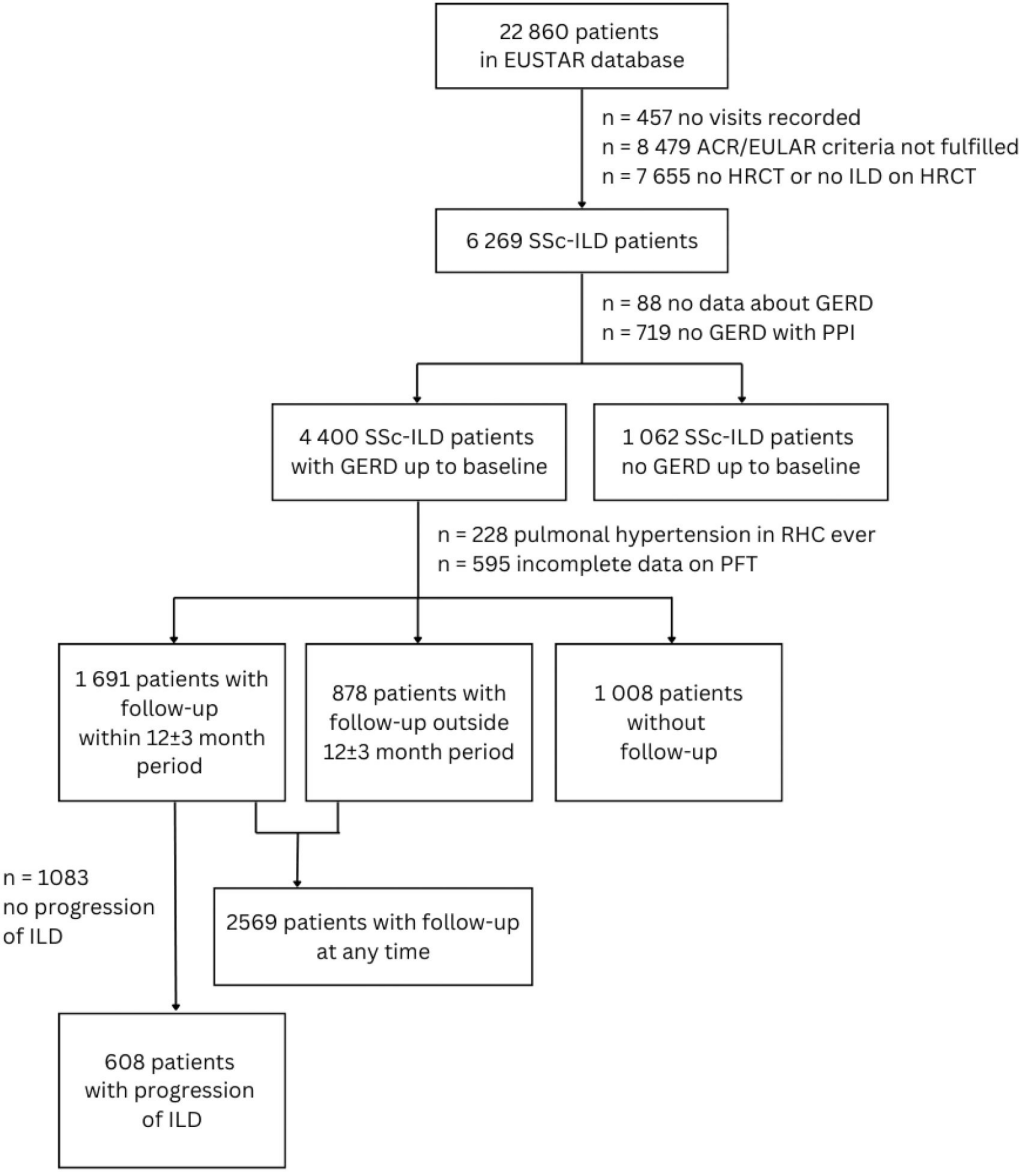
Flow-chart of study process. Progression of ILD if [1] FVC decline ≥ 10% or [2] FVC decline 5–9% in association with DLCO decline ≥15% in 12±3 months [26]. ACR/EULAR: American College of Rheumatology/European League against rheumatism; GERD: gastroesophageal reflux disease; HRCT: high-resolution computed tomography; ILD: interstitial lung disease; PFT: pulmonary function tests; PPI: proton pump inhibitors; RHC: right heart catheter; SSc: systemic sclerosis

We performed additional exploratory analyses. We assessed SSc-ILD patients with GERD with any available follow-up but not within annual range. In addition, we examined our study outcomes in SSc-ILD patients with GERD who reported active esophageal symptoms at baseline visit (for flow-chart, see Fig. 1).

### Statistical analysis

Categorical variables are presented in absolute numbers and percentages, whereas continuous variables are described using mean and standard deviation. Cross-sectional analysis was performed using independent *t* test or χ^2^ test according to the distribution of the variable to compare the GERD and non-GERD subpopulations at baseline, as well as patients with or without PPI use in the whole SSc-ILD population. To identify disease characteristics independently associated with GERD, a multivariable logistic regression model was applied obtaining OR and 95% confidence intervals (CI). The different variables were tested for collinearity. A high collinearity was defined by r > 0.7 [29]. The area under the curve (AUC) and 95% CI of the logistic regression model were calculated.

A multivariable Cox proportional hazard regression model (with hazard ratios HR and 95% CI) was performed to identify the predictive factors for ILD progression.

Covariates for the multivariable models were selected according to literature and expert opinion. These included sex, age, disease duration, cutaneous subset according to LeRoy classification, smoking status, anti-topoisomerase 1 antibodies, FVC, DLCO, dyspnea NYHA class >2, use of ILD modifying treatment and use of PPI [2, 5, 6]. To account for missing values, the data for multivariable models (logistic and cox regression) were analysed using multiple imputations by chained equations with 10 imputations after 10 iterations [30, 31].

Progression-free survival and overall survival were assessed using the Kaplan–Meier method.

The significance level for all tests was two-sided and set at 0.05. Statistical analysis was performed using IBM SPSS Version 29.0.0.0.

## Results

### Baseline characteristics

Among 22 860 patients in the EUSTAR cohort, 5462 SSc-ILD patients were included in the current study. Overall, 4400 (80.6%) patients reported GERD symptoms and 1062 (19.4%) no GERD symptoms before or at baseline (flow-chart in Fig. 1).

At baseline, SSc-ILD patients with GERD were more often female (83.8% *vs* 80.0%, *P* = 0.003), had longer disease duration (10.2 ± 8.8 *vs* 7.1 ± 7.4 years, *P < *0.001) and more frequently the diffuse cutaneous subset (50.2% *vs* 38.7%, *P < *0.001) as compared with SSc-ILD patients without GERD. SSc-ILD patients with GERD had more often other gastrointestinal symptoms, musculoskeletal involvement, left heart dysfunction and vascular involvement as reflected by higher frequencies of digital ulcers, telangiectasia and late scleroderma pattern on capillaroscopy. Disease characteristics of SSc-ILD patients with and without GERD are presented in Table 1.

**Table 1.** Baseline characteristics of the GERD and non-GERD SSc-ILD subpopulations

	Total	GERD^a^	non GERD^a^	N data available	*P* value*
	*n* = 5462	*n* = 4400	*n* = 1062		
Female sex	4538/5461 (83.1)	3688/4399 (83.8)	850/1062 (80.0)	5461	0.003
Age at baseline, years	57.1 (13.3)	57.3 (13.2)	56.6 (13.7)	5462	0.133
Ethnicity				4952	<0.001
White	4513/4952 (91.1)	3651/3984 (91.6)	862/968 (89.0)		
Asian	210/4952 (4.2)	147/3984 (3.7)	63/968 (6.5)		
Black	76/4952 (1.5)	60/3984 (1.5)	16/968 (1.7)		
Other	153/4952 (3.1)	126/3984 (3.2)	27/968 (2.8)		
Ever smoker	1405/3971 (35.4)	1147/3276 (35.0)	258/695 (37.1)	3971	0.291
Disease duration, years	9.7 (8.7)	10.2 (8.8)	7.1 (7.4)	4697	<0.001
Diffuse cutaneous SSc^b^^,^^c^	2003/4174 (48.0)	1695/3378 (50.2)	308/796 (38.7)	4174	<0.001
SSc^b^-specific auto-antibodies^c^					
Anti-Centromere	980/4761 (20.6)	798/3837 (20.8)	182/924 (19.7)	4761	0.458
Anti-Topoisomerase 1	2540/4886 (52.0)	2043/3936 (51.9)	497/950 (52.3)	4886	0.820
Anti-RNA Polymerase III	171/3362 (5.1)	137/2717 (5.0)	34/645 (5.3)	3362	0.812
Treatment					
ILD modifying treatment^d^	1492/3912 (38.1)	1308/3230 (40.5)	184/682 (27.0)	3912	<0.001
Corticosteroids	1355/3912 (34.6)	1216/3230 (37.6)	139/682 (20.4)	3912	<0.001
Prednisone dose <10 mg/day	964/1248 (77.2)	878/1121 (78.3)	86/127 (67.7)	1248	0.007
Prednisone dose, mg	7.4 (7.3)	7 (5.8)	10.7 (15.0)	640	0.061
Proton pump inhibitors	1987/3912 (50.8)	1987/3230 (61.5)	0/682 0	3912	<0.001
CRP^e^ elevation >5 mg/L	1089/5367 (20.3)	897/4321 (20.8)	192/1046 (18.4)	5367	0.083
Scleroderma renal crisis	90/5371 (1.7)	72/4328 (1.7)	18/1043 (1.7)	5371	0.888
Scleroderma pattern at capillaroscopy	2621/2897 (90.5)	2101/2307 (91.1)	520/590 (88.1)	2897	0.030
Early scleroderma pattern	421/2055 (20.5)	315/1647 (19.1)	106/408 (26)	2055	
Active scleroderma pattern	801/2055 (39)	613/1647 (37.2)	188/408 (46.1)	2055	
Late scleroderma pattern	833/2055 (40.5)	719/1647 (43.7)	114/408 (27.9)	2055	
Digital ulcers				3897	<0.001
Current	704/3897 (18.1)	605/3198 (18.9)	99/699 (14.2)		
Previously	1233/3897 (31.6)	1080/3198 (33.8)	153/699 (21.9)		
Never	1960/3897 (50.3)	1513/3198 (47.3)	447/699 (63.9)		
Telangiectasia	2429/3895 (62.4)	2070/3195 (64.8)	359/700 (51.3)	3895	<0.001
Heart dysfunction (LVEF^f^ <50%)	358/4653 (7.7)	312/3746 (8.3)	46/907 (5.1)	4653	<0.001
Stomach symptoms^g^	1238/5347 (23.2)	1174/4299 (27.3)	64/1048 (6.1)	5347	<0.001
Intestinal symptoms^g^	1336/5361 (24.9)	1202/4312 (27.9)	134/1049 (12.8)	5361	<0.001

Variables are presented as *n* (percentages) or as means (SD).

a GERD, gastroesophageal reflux disease.

b SSc, systemic sclerosis.

c Assessment of cutaneous subset according LeRoy classification [23].

d ILD (interstitial lung disease) modifying treatment includes cyclophosphamide, mycophenolate mofetil, tocilizumab, rituximab, nintedanib, autologous stem cell transplantation and lung transplantation [25].

e CRP, c-reactive protein.

f LVEF, left ventricular ejection fraction.

g Stomach and intestinal symptoms were defined by past medical history.

* *P* values were obtained using students *t* test or χ^2^ test.

Lung involvement was more severe in SSc-ILD patients with GERD as compared with those without GERD, as reflected by more respiratory symptoms, a lower FVC%pred (85.8 ± 22.1 *vs* 90.2 ± 20.1, *P < *0.001), a lower DLCO%pred (60.8 ± 19.7 *vs* 65.3 ± 20.6, *P < *0.001), worse performance at the 6 min walking test and more frequent use of ILD modifying treatment (40.5% *vs* 27.0%, *P < *0.001) (Table 2).

**Table 2.** Characteristics of lung involvement in SSc-ILD patients with and without GERD

	Total	GERD^a^	Non GERD^a^	N data available	*P* value*
	*n* = 5462	*n* = 4400	*n* = 1062		
Dyspnea NYHA^b^ >2	714/4901 (14.6)	616/3956 (15.6)	98/945 (10.4)	4901	<0.001
O2-saturation at rest	96.6 (4.0)	96.5 (4.1)	97.1 (3.7)	1492	0.018
FVC%pred^c^ at baseline	86.6 (21.8)	85.8 (22.1)	90.2 (20.1)	4637	<0.001
FVC%pred^c^ <80%	1740/4637 (37.5)	1474/3729 (39.5)	266/908 (29.3)	4637	<0.001
DLCO%pred^d^ at baseline	61.7 (20.0)	60.8 (19.7)	65.3 (20.6)	4286	<0.001
DLCO%pred^d^ <70%	2844/4286 (66.4)	2335/3447 (67.7)	509/839 (60.7)	4286	<0.001
6-min walking distance, metres	438.8 (131.3)	431.5 (132.9)	466.2 (121.3)	1576	<0.001

Variables are presented as *n* (percentages) or as means (SD).

a GERD, gastroesophageal reflux.

b NYHA, New York Heart Association [24].

c FVC%pred, % predicted forced vital capacity.

d DLCO%pred, % predicted diffusing capacity of lungs for carbon monoxide.

* *P*-value obtained using χ^2^ tests or students t-tests.

A multivariable logistic regression analysis was performed to identify independent factors associated with GERD in SSc-ILD patients. A longer disease duration (OR: 1.05 [1.04–1.06], *P < *0.001), diffuse cutaneous SSc (OR: 1.44 [1.22–1.69], *P < *0.001), stomach symptoms (OR: 4.44 [3.41–5.79], *P < *0.001) and intestinal symptoms (OR: 1.87 [1.52–2.30], *P < *0.001) were depicted as independent risk factors. Furthermore, lower DLCO%pred at baseline (OR: 0.99 [0.99–1.00], *P* = 0.015) and treatment with ILD-modifying drugs (OR: 1.49 [1.25–1.78], *P < *0.001) were also independently associated with GERD in SSc-ILD patients (Table 3). Correlations between predictor variables were low (r < 0.50), indicating that multicollinearity was not a biasing factor in the analysis [29].

**Table 3.** Factors independently associated with GERD in SSc-ILD subpopulation

Covariates	OR [95% CI]	*P* value*
Male sex	0.90 [0.74–1.10]	0.294
Age at baseline, years	1.00 [1.00–1.01]	0.332
**Disease duration, years**	**1.05 [1.04**–**1.06]**	**<0.001**
**Diffuse cutaneous SSc** ^a^	**1.44 [1.22**–**1.69]**	**<0.001**
Anti-Centromere^b^	1.17 [0.94–1.45]	0.154
Ever smoker	0.92 [0.77–1.10]	0.348
CRP^c^ elevation >5 mg/L	1.02 [0.85–1.23]	0.817
Dyspnea NYHA^d^ >2	1.17 [0.92–1.50]	0.204
FVC%pred^e^ at baseline	1.00 [0.99–1.00]	0.105
**DLCO%pred** ^f^ **at baseline**	**0.99 [0.99**–**1.00]**	**0.015**
**Stomach symptoms** ^g^	**4.44 [3.41**–**5.79]**	**<0.001**
**Intestinal symptoms** ^g^	**1.87 [1.52**–**2.30]**	**<0.001**
**ILD modifying treatment** ^h^	**1.49 [1.25**–**1.78]**	**<0.001**

a SSc, systemic sclerosis.

b Assessment of cutaneous subset according LeRoy classification [23].

c CRP, C-reactive protein.

d NYHA, New York Heart Association [24].

e FVC%pred, % predicted forced vital capacity.

f DLCO%pred, % predicted diffusing capacity of lungs for carbon monoxide.

g Stomach and intestinal symptoms were defined by past medical history.

h ILD (interstitial lung disease) modifying treatment includes cyclophosphamide, mycophenolate mofetil, tocilizumab, rituximab, nintedanib, autologous stem cell transplantation and lung transplantation [25].

* *P* values were obtained using multivariable logistic regression with multiple imputations. Bold text highlights significant results.

PPI were reported as ongoing in 1987/3230 (61.5%) GERD patients at baseline visit. SSc-ILD patients with GERD currently treated with PPI had a more severe ILD disease than patients without current use of PPI, with also a more frequent use of ILD modifying treatment (48.7% *vs* 27.4%, *P < *0.001), more frequently dyspnea with NYHA >2 (18.4% *vs* 12.2%, *P < *0.001), lower values of FVC%pred (83.8 ± 22.7 *vs* 87.5 ± 21.6, *P < *0.001), DLCO%pred (58.6 ± 19.5 *vs* 61.4 ± 19.5, *P < *0.001) and 6 min walking distance (Supplementary Table S1, available at *Rheumatology* online).

### Longitudinal analysis

Over a mean follow-up of 6.0 ± 4.0 years, 1691 SSc-ILD patients with GERD with at least one annual follow-up visit could be included in the longitudinal analysis (flow-chart in Fig. 1). An average of 2.8 ± 2.2 annual intervals were studied. Patients with annual visits had less severe lung involvement compared with patients with follow-ups outside annual range; however, the mortality rate did not differ significantly (Supplementary Table S2, available at *Rheumatology* online).

Among the 1691 SSC-ILD patients, 608 (35.9%) patients had at least one progression episode of ILD and this event occurred on average 3.5 ± 3.03 years after baseline visit.

Over a mean follow-up of 6.0 ± 4.0 years, 192 of 1691 (11.4%) SSc-ILD patients with GERD died, of which 90 (46.9%) patients had previous progression of ILD. Overall, 710/1691 (41.9%) experienced ILD progression and/or death. There was no significant difference in progression-free survival in GERD and non-GERD subpopulations (Supplementary Fig. S1, available at *Rheumatology* online).

Because we labelled GERD as patients with gastroesophageal symptoms before and/or at baseline visit, we additionally analysed the subgroup with active gastroesophageal symptoms at baseline visit. This subpopulation was a consistent part of the whole group, showing an active involvement in 88% of SSc-ILD patients with GERD (3834/4352), with longitudinal data for 1454 patients. Over a mean follow-up of 6.0 ± 4.0 years including these 1454 GERD patients with active symptoms, progression of ILD occurred on average after 3.6 ± 3.0 years in totally 531 patients, in line with the numbers of the whole group.

### Predictors of progression, mortality and progression-free survival in the whole SSc-ILD population with GERD

In SSc-ILD patients with GERD, female sex (HR: 1.39 [1.07–1.80], *P* = 0.012) and older age at baseline (HR: 1.02 [1.01–1.03], *P < *0.001) independently predicted progression of ILD (Table 4). Consistently, relative FVC%pred decline ≥10% was more prevalent in females than in males (31.9% *vs* 25%, *P* = 0.020), without significant difference regarding DLCO%pred decline (Supplementary Table S3, available at *Rheumatology* online).

**Table 4.** Predictive factors for progression of ILD in longitudinal SSc-ILD subpopulation with GERD

	Progression of ILD^a^ over 12 ± 3 months	
Covariates	HR [95% CI]	*P* value*
Female sex	**1.39 [1.07**–**1.80]**	**0.012**
Age at baseline, years	**1.02 [1.01**–**1.03]**	**<0.001**
Disease duration, years	0.99 [0.98–1.00]	0.099
Diffuse cutaneous SSc^b^^,^^c^	1.08 [0.88–1.33]	0.469
Anti-Topoisomerase 1^c^	0.96 [0.79–1.18]	0.725
Ever smoker	1.01 [0.76–1.34]	0.964
Dyspnea NYHA^d^ >2	1.16 [0.89–1.52]	0.265
FVC%pred^e^ at baseline	1.00 [0.99–1.00]	0.097
DLCO%pred^f^ at baseline	1.00 [0.99–1.00]	0.305
ILD modifying treatment^g^	1.06 [0.86–1.31]	0.603
Proton pump inhibitors	0.96 [0.78–1.18]	0.693

a ILD, interstitial lung disease.

b SSc, systemic sclerosis.

c Assessment of cutaneous subset according LeRoy classification [23].

d NYHA, New York Heart Association [24].

e FVC%pred, % predicted forced vital capacity.

f DLCO%pred, % predicted diffusing capacity of lungs for carbon monoxide.

g ILD (interstitial lung disease) modifying treatment includes cyclophosphamide, mycophenolate mofetil, tocilizumab, rituximab, nintedanib, autologous stem cell transplantation and lung transplantation [25].

* *P* values were obtained using cox regression analyses with multiple imputations. Bold text highlights significant results.

Predictive factors for mortality were older age (HR: 1.06 [1.05–1.08], *P < *0.001), diffuse cutaneous subset (HR: 1.49 [1.05–2.11], *P* = 0.026), and lower FVC%pred (HR: 0.99 [0.98–1.00], *P* = 0.040) and lower DLCO%pred at baseline (HR: 0.98 [0.96–0.99], *P < *0.001) (Table 5).

**Table 5.** Predictive factors for overall survival and progression-free survival in longitudinal SSc-ILD subpopulation with GERD

	Death		Progression of ILD^a^ over 12 ± 3 months or death	
	*n* = 192		*n* = 710	
Covariates	HR [95% CI]	*P* value	HR [95% CI]	*P* value
Female sex	0.73 [0.50–1.08]	0.119	**1.32 [1.04**–**1.66]**	**0.021**
Age at baseline, years	**1.06 [1.05**–**1.08]**	**<0.001**	**1.02 [1.01**–**1.03]**	**<0.001**
Disease duration, years	1.01 [0.99–1.03]	0.355	0.99 [0.98–1.00]	0.235
Diffuse cutaneous SSc^b^^,^^c^	**1.49 [1.05**–**2.11]**	**0.026**	1.11 [0.90–1.36]	0.319
Anti-Topoisomerase 1^c^	1.18 [0.81–1.72]	0.380	0.94 [0.77–1.15]	0.553
Ever smoker	1.34 [0.90–2.00]	0.143	1.03 [0.80–1.33]	0.833
Dyspnea NYHA^d^ >2	1.23 [0.80–1.90]	0.345	1.16 [0.91–1.49]	0.224
FVC%pred^e^ at baseline	**0.99 [0.98**–**1.00]**	**0.040**	**0.99 [0.99**–**1.00]**	**0.030**
DLCO%pred^f^ at baseline	**0.98 [0.96**–**0.99]**	**<0.001**	1.00 [0.99–1.00]	0.105
ILD modifying treatment^g^	0.74 [0.49–1.10]	0.139	1.02 [0.83–1.25]	0.856
Proton pump inhibitors	1.17 [0.76–1.79]	0.462	0.98 [0.80–1.20]	0.825

a ILD, interstitial lung disease.

b SSc, systemic sclerosis.

c Assessment of cutaneous subset according LeRoy classification [23].

d NYHA, New York Heart Association [24].

e FVC%pred, % predicted forced vital capacity.

f DLCO%pred, % predicted diffusing capacity of lungs for carbon monoxide.

g ILD modifying treatment includes cyclophosphamide, mycophenolate mofetil, tocilizumab, rituximab, nintedanib, autologous stem cell transplantation and lung transplantation [25].

* *P* values were obtained using cox regression analyses with multiple imputations. Bold text highlights significant results.

Predictive factors for progression of ILD or death in SSc-ILD patients with GERD were female sex (HR: 1.32 [1.04–1.66], *P* = 0.021), older age (HR: 1.02 [1.01–1.03], *P < *0.001) and lower FVC%pred at baseline (Table 5).

In the exploratory subanalyses including GERD patients with active esophageal symptoms at baseline female, older age and lower FVC%pred at baseline were confirmed predictive for progression of ILD (Supplementary Table S4, available at *Rheumatology* online). Older age and lower DLCO%pred predicted mortality in this subgroup of GERD patients with active symptoms at baseline. Predictive factors for progression of ILD or death were similar to the whole SSc-ILD subpopulation with GERD (Supplementary Table S5, available at *Rheumatology* online). To determine whether females generally have a higher risk of ILD progression or if this is specific to the subgroup of SSc-ILD patients with GERD, we assessed risk factors for ILD progression in the entire SSc-ILD cohort. In this cohort, female sex was also identified as a predictor of ILD progression (HR: 1.22 [1.00–1.49], *P* = 0.046) (Supplementary Table S6, available at *Rheumatology* online). However, 80% of this cohort had GERD. An additional subanalysis was therefore performed on the non-GERD cohort without PPI use, representing the pure non-GERD subgroup. In this subgroup, female sex was no longer a predictive factor for ILD progression (Supplementary Table S7, available at *Rheumatology* online), suggesting that the predictive factors for ILD progression may vary, particularly with regard to sex, depending on the presence of GERD.

### Exploratory analyses of survival according to PPI usage

In our exploratory analysis, 2569 SSc-ILD GERD patients with at least one follow-up at any time (flow-chart in Fig. 1) were included and analysed regarding mortality in up to 8 years follow-up (mean follow-up 4.8 ± 3.9 years). The overall survival of patients with current use of PPI significantly differed from patients without PPI (*P* = 0.022) (Supplementary Fig. S2, available at *Rheumatology* online).

## Discussion

To our knowledge, this is the first study to specifically characterize the phenotype of SSc-ILD patients with GERD. In our cohort, more than three quarters of SSc-ILD patients had GERD symptoms, consistent with previous data [12, 14].

We were able to precisely define the characteristics of SSc-ILD patients with GERD: they had overall a more severe ILD phenotype, as reflected by the higher prevalence of respiratory symptoms, lower FVC%pred and DLCO%pred values, worse performance on the 6-min walking test and more frequent use of ILD modifying treatment. Moreover, they were characterized by a more severe systemic disease with more diffuse cutaneous subset, left heart dysfunction, inflammatory and vascular manifestations, suggesting that the presence of GERD may characterize SSc-ILD patients with a more severe lung involvement but also with a more severe SSc overall. Consistently, the use of PPI as a surrogate marker for more severe and possibly active GERD showed an association of a more severe lung involvement in GERD patients with PPI treatment at the time of the consultation. These data confirm a previous study of the German cohort on 1931 SSc-ILD patients, where PPI use was associated with lower FVC, lower DLCO and more frequent use of immunosuppressive therapies [16]. Therefore, the presence of GERD and the use of PPI might help to risk stratify SSc-ILD patients towards a more severe disease.

In our study, SSc-ILD patients with GERD and PPI use showed poorer survival, suggesting that patients with a more severe GERD needing persistent use of PPI have worse outcomes. Conversely in the German cohort, the use of PPI prevented ILD progression [16]. However, the German study included fewer patients as compared with ours (1050 *vs* 2472 patients). In addition, in both cohorts, the date on which PPI were prescribed and the duration of treatment were not specified, which may explain the discrepancies. One may hypothesize that treatment started at early stages of the ILD or before ILD diagnosis has an impact on ILD course. However, in a cohort study, gastroprotective agents did not prevent the onset of SSc-ILD [32]. Therefore, the effect of PPI to prevent progression of ILD in SSc-ILD patients with GERD remains mostly unclear. An update of the 2022 guideline for IPF has removed PPIs as a treatment option due to insufficient data to support benefit [27]. Therefore, prospective, randomized, controlled clinical trials are needed to assess the effects of PPI use in SSc-ILD. To select the population to enrich in this trial, the factors associated with ILD progression in this subpopulation must be identified. In our study, female sex and older age were identified as risk factors for progression of ILD in the SSc-ILD subpopulation with GERD. Although male sex was associated with development of ILD in SSc [33], the impact of sex on the progression of ILD remains less clear [5, 17, 34]. In a study by Le Gouellec *et al.*, sex has not been identified as a predictor for progression of ILD; however, the results were limited by the small sample size of the cohort (*n* = 75) including only 18 male patients [34]. In a EUSTAR study by Hoffmann-Vold *et al.* [5], male sex together with reflux were amongst the strongest predictive factors for FVC decline over 5 years. These discordant results in the EUSTAR cohort may be explained by a smaller sample size (826 *vs* 1691), different inclusion criteria and the adjustment of the models for the presence of reflux. On the other hand, our results are consistent with a recent EUSTAR study by Campochiaro *et al.* which shows that women more frequently experienced progression of ILD [17]. To determine whether females generally have a higher risk of ILD progression or if this is specific to the subgroup of SSc-ILD patients with GERD, we assessed risk factors for ILD progression in the non-GERD cohort without PPI use, representing the pure non-GERD subgroup. In this subgroup, female sex was no longer a predictive factor for ILD progression, suggesting that female sex could represent a new risk factor in the SSc-ILD population with GERD. This data needs to be considered for further cohort enrichment for clinical trials. Our results also suggest the development of a sex-stratified approach in SSc-ILD patients with GERD. One possible explanation for this sex difference could be anatomical factors, as men physiologically have a longer esophagus than women, which may reduce their susceptibility to microaspirations that contribute to the progression of lung fibrosis [35]. Another known risk factor for progression of ILD in the general SSc-ILD population is the presence of anti-topoisomerase 1 antibodies, which does not appear to play a significant role in progression of ILD in our SSc-ILD population with GERD, confirming a previous study by Zhang *et al.* [36].

Despite the substantial differences and more severe organ involvement in SSc-ILD patients with GERD, no significant difference in progression-free survival was observed between the GERD and non-GERD subpopulations in our analysis. These results are consistent with the study by Kreuter *et al.* [16]. However, GERD patients with active reflux, as indicated by the use of PPIs, exhibited more severe lung involvement and poorer overall survival in a sub-analysis of the SSc-ILD-GERD group.

Our study should be interpreted within its limitations. Our definition of GERD was based on a history of reflux and/or dysphagia symptoms and severity of GERD symptoms was not recorded [37]. To date, the diagnosis of GERD can be made both symptom-based and by physiologic testing and is limited in both respects; due to the pragmatic approach to clinical practice, the diagnosis is often made clinically.

A study by Volkmann *et al.* showed a clear correlation of severity of reflux symptoms with progressive ILD using SSc-specific questionnaires; in contrast, no association could be found with radiographically measured esophageal diameters and ILD progression [15]. Thus, it could be hypothesized that patient-oriented questioning has a greater benefit in determining severity than instrument-based measurements. Although such a scoring system was not available in our study, we approximated reflux severity by current use of PPI as a surrogate marker of more severe reflux disease. However, it must be acknowledged that the persistent use of PPIs may not be a marker of GERD severity, but rather an indicator of ongoing active reflux. Gastric ulcer disease could potentially serve as a marker for assessing reflux severity. Unfortunately, peptic ulcer disease was not examined in our study, as gastroscopy results were unavailable in the EUSTAR database. Furthermore, it could be argued that we only proceeded according to the symptom-based GERD diagnosis, whereby only upper gastrointestinal complaints before and/or at baseline visit were surveyed. To ensure that the current symptom burden is also examined, we included a subanalysis investigating active reflux symptoms at baseline. As expected, this did not show any major difference to our main results. In addition, it must be taken into account that the use of medications such as calcium channel blockers or endothelin receptor antagonists can also promote reflux, and it would be difficult to distinguish between GERD due to SSc disease and a possible drug side effect based on the symptoms alone. Due to a high number of missing data on the use of these medications at baseline (>60%), this could not be studied in the present analysis. However, the prevalence of GERD observed in our cohort was consistent with previous reports in SSc and SSc-ILD [12–15, 38, 39].

For the definition of progression of ILD, we used the composite functional criteria and stringent definition with considerable decrease of FVC, as suggested by Goh *et al.* [26]. However, it should be noted that this definition only includes pulmonary functional tests and neither clinical symptoms nor measurements of the radiologic extent or pattern of the lung disease are taken into account, as for instance in the ATS/ERS criteria [27]. However, a limited number of HRCTs were performed in our cohort, and the extent of fibrosis was only estimated as > and <20% in the EUSTAR database, which is inadequate for further analysis. Furthermore, data regarding respiratory symptoms at follow-up were missing, which is why the application of these classification criteria was not feasible. There are a variety of different criteria for progression of ILD, which makes comparability between studies difficult. To validate our results, further studies using different criteria for progression of ILD would be necessary.

Another limiting factor is not considering only deaths of pulmonary origin. The cause of death was unknown in our cohort. However, the precise cause of death can be difficult to determine, and ILD, even if it is sometimes not the direct cause of death, can often precipitate/favor the death in this context (e.g. death due to lung infection, etc). In this way, death reflects the overall severity of the disease, which is largely explained by lung damage in SSc. Furthermore, a limitation may be related to the presence of missing data in the EUSTAR registry. However, <8% of the data were missing, and the presence of missing data was addressed by performing multiple imputations. Finally, most patients were of Caucasian descent, therefore study results additionally need to be validated for other ethnicities.

Nonetheless, our study also has important strengths. This is the first study to evaluate characteristics and disease progression in SSc-ILD patients with GERD in such a large number of patients. Multivariable models were done to determine accurately the risk factors for ILD progression. Additionally, for our longitudinal analysis we excluded patients with pulmonal hypertension at any time point diagnosed in RHC, because DLCO values are altered in pulmonary hypertension [2]. Moreover, to avoid the confounding factor, patients without reported GERD symptoms before and/or at the time of the consultation but using PPI were excluded from further analyses.

In conclusion, GERD is a common manifestation in SSc-ILD and is associated with more severe disease. Predictive factors for ILD progression in patients with GERD may differ from the general SSc-ILD population, with a possible higher risk in women.

## Supplementary material

Supplementary material is available at *Rheumatology* online.

## Supplementary Material

keaf016_Supplementary_Data

## Data Availability

Data are owned by EUSTAR and can be made available upon request.
