# Supplementary material for: Gastroesophageal reflux disease is associated with a more severe interstitial lung disease in systemic sclerosis in the EUSTAR cohort
Source: Rheumatology (Oxford). 2025 Jan 8;64(SI):SI63–72. doi: 10.1093/rheumatology/keaf016 (PMC12695048; doi:10.1093/rheumatology/keaf016)
Supplement: keaf016_Supplementary_Data [file keaf016_supplementary_data.zip › e54be_rhe-24-2408-File002.docx]

**SUPPLEMENTARY MATERIAL**

**Supplementary Data S1**

STROBE Statement—Checklist of items that should be included in reports of ***cohort studies***

|  | **Item No** | **Recommendation** |
| --- | --- | --- |
| **Title and abstract** | 1 | (*a*) Indicate the study’s design with a commonly used term in the title or the abstract  *Study design is described in the abstract* |
|  |  | (*b*) Provide in the abstract an informative and balanced summary of what was done and what was found  *Provided in the abstract* |
| **Introduction** | | |
| Background/rationale | 2 | Explain the scientific background and rationale for the investigation being reported  *Scientific background and rationale are explained in the introduction* |
| Objectives | 3 | State specific objectives, including any prespecified hypotheses  *Stated at the end of introduction section* |
| **Methods** | | |
| Study design | 4 | Present key elements of study design early in the paper  Presented early in method section |
| Setting | 5 | Describe the setting, locations, and relevant dates, including periods of recruitment, exposure, follow-up, and data collection  *Presented in method section* |
| Participants | 6 | (*a*) Give the eligibility criteria, and the sources and methods of selection of participants. Describe methods of follow-up  *This is described in method section* |
|  |  | (*b*) For matched studies, give matching criteria and number of exposed and unexposed  *NA* |
| Variables | 7 | Clearly define all outcomes, exposures, predictors, potential confounders, and effect modifiers. Give diagnostic criteria, if applicable  *This is described in method section* |
| Data sources/ measurement | 8* | For each variable of interest, give sources of data and details of methods of assessment (measurement). Describe comparability of assessment methods if there is more than one group  *This is described in method section* |
| Bias | 9 | Describe any efforts to address potential sources of bias  *This is described in the method section (exclusion of patients with use of proton pomp inhibitors and patients with pulmonary hypertension at any time)* |
| Study size | 10 | Explain how the study size was arrived at  *This is described in method section and showed in the flowchart (Figure 1)* |
| Quantitative variables | 11 | Explain how quantitative variables were handled in the analyses. If applicable, describe which groupings were chosen and why  *This is described in the statistical analysis subsection of method section* |
| Statistical methods | 12 | (*a*) Describe all statistical methods, including those used to control for confounding  *This is described in the statistical analysis subsection of method section* |
|  |  | (*b*) Describe any methods used to examine subgroups and interactions  *This is described in the statistical analysis subsection of method section* |
|  |  | (*c*) Explain how missing data were addressed  *To account for missing observations, the data for multivariate models (logistic and cox regression) were analyzed using multiple imputations by chained equations, with 10 imputations obtained after 10 iterations* |
|  |  | (*d*) If applicable, explain how loss to follow-up was addressed  *Patients lost to follow-up were not included in the longitudinal analysis. Patients lost to follow-up were compared in Supplementary Table S3* |
|  |  | (*e*) Describe any sensitivity analyses  *Sensitivity analyses were performed and are described in method section* |
| **Results** | | |
| Participants | 13* | (a) Report numbers of individuals at each stage of study—e.g. numbers potentially eligible, examined for eligibility, confirmed eligible, included in the study, completing follow-up, and analyzed  *Reported in results section and flow chart (Figure 1)* |
|  |  | (b) Give reasons for non-participation at each stage  *Reported in results section and flow chart (Figure 1)* |
|  |  | (c) Consider use of a flow diagram  *Figure 1* |
| Descriptive data | 14* | (a) Give characteristics of study participants (eg demographic, clinical, social) and information on exposures and potential confounders  *Reported in results section and shown in Table 1* |
|  |  | (b) Indicate number of participants with missing data for each variable of interest  *Reported in results section, Table 1* |
|  |  | (c) Summarize follow-up time (e.g. average and total amount)  *Reported in results section* |
| Outcome data | 15* | Report numbers of outcome events or summary measures over time  *Reported in results section* |
| Main results | 16 | (*a*) Give unadjusted estimates and, if applicable, confounder-adjusted estimates and their precision (e.g., 95% confidence interval). Make clear which confounders were adjusted for and why they were included  *Reported in results section and in tables* |
|  |  | (*b*) Report category boundaries when continuous variables were categorized  *Reported in results section and in tables* |
|  |  | (*c*) If relevant, consider translating estimates of relative risk into absolute risk for a meaningful time period  *NA* |
| Other analyses | 17 | Report other analyses done—e.g. analyses of subgroups and interactions, and sensitivity analyses  *Reported in results section and in Supplementary Tables* |
| **Discussion** | | |
| Key results | 18 | Summarize key results with reference to study objectives  *Reported in the discussion section* |
| Limitations | 19 | Discuss limitations of the study, taking into account sources of potential bias or imprecision. Discuss both direction and magnitude of any potential bias  *Limitations are discussed in the discussion section* |
| Interpretation | 20 | Give a cautious overall interpretation of results considering objectives, limitations, multiplicity of analyses, results from similar studies, and other relevant evidence  *Limitations are discussed in the discussion section* |
| Generalizability | 21 | Discuss the generalizability (external validity) of the study results  *These points are discussed in the discussion section* |
| **Other information** | | |
| Funding | 22 | Give the source of funding and the role of the funders for the present study and, if applicable, for the original study on which the present article is based  *There was no funding for this study.* |

*Give information separately for exposed and unexposed groups.

**Note:** An Explanation and Elaboration article discusses each checklist item and gives methodological background and published examples of transparent reporting. The STROBE checklist is best used in conjunction with this article (freely available on the Web sites of PLoS Medicine at http://www.plosmedicine.org/, Annals of Internal Medicine at http://www.annals.org/, and Epidemiology at http://www.epidem.com/). Information on the STROBE Initiative is available at <http://www.strobe-statement.org>.

|  | Total  n = 3230 | GERD^a^ with PPI  n = 1987 | GERD^a^ without PPI  n = 1243 | N data available | p value* |
| --- | --- | --- | --- | --- | --- |
| Female sex | 2680/3229  (83.0) | 1665/1986  (83.8) | 1015/1243  (81.7) | 3229 | 0.109 |
| Age at baseline, years | 57.9  (13.2) | 57.6  (13.2) | 58.4  (13.3) | 3230 | 0.104 |
| Ever smoker | 1060/3035 (34.9) | 640/1852  (34.6) | 420/1183  (35.5) | 3035 | 0.594 |
| Disease duration, years | 10.2  (9.0) | 9.7  (8.8) | 11.0  (9.3) | 2769 | <0.001 |
| Disease duration > 5 years | 1820/2769 (65.7) | 1089/1717  (63.4) | 731/1052  (69.5) | 2769 | 0.001 |
| Diffuse cutaneous SSc^bc^ | 1415/2761 (51.2) | 905/1726  (52.4) | 510/1035  (49.3) | 2761 | 0.108 |
| Anti-Centromere^c^ | 576/2755  (20.9) | 329/1689  (19.5) | 247/1066  (23.2) | 2755 | 0.020 |
| Anti-Topoisomerase 1^c^ | 1485/2863 (51.9) | 939/1771  (53) | 546/1092  (50.0) | 2863 | 0.116 |
| Anti-RNA  Polymerase III^c^ | 122/2011  (6.1) | 75/1219  (6.2) | 47/792  (5.9) | 2011 | 0.841 |
| Treatment | | | | | |
| ILD modifying treatment^d^ | 1308/3230 (40.5) | 968/1987  (48.7) | 340/1243  (27.4) | 3230 | <0.001 |
| Corticosteroids | 1216/3230 (37.6) | 991/1987  (49.9) | 225/1243  (18.1) | 3230 | <0.001 |
| Prednisone dose  < 10 mg/day | 878/1121  (78.3) | 728/920  (79.1) | 150/201  (74.6) | 1121 | 0.160 |
| Prednisone dose, mg | 7  (5.8) | 7  (5.9) | 7.2  (4.9) | 578 | 0.791 |
| Proton pump inhibitors | 1987/3230 (61.5) | 1987/1987  (100) | 0/1243  (0) | 3230 | <0.001 |
| Lung characteristics | | | | | |
| Dyspnea NYHA^e^ > 2 | 466/2895  (16.1) | 332/1801  (18.4) | 134/1094  (12.2) | 2895 | <0.001 |
| O2-saturation at rest | 96.6  (4.0) | 96.5  (3.5) | 96.6  (4.7) | 950 | 0.721 |
| FVC%pred^f^ at baseline | 85.2  (22.3) | 83.8  (22.7) | 87.5  (21.6) | 2704 | <0.001 |
| FVC%pred^f^ < 80% | 1107/2704 (40.9) | 732/1685  (43.4) | 375/1019  (36.8) | 2704 | <0.001 |
| DLCO%pred^g^ at baseline | 59.6  (19.6) | 58.6  (19.5) | 61.4  (19.5) | 2434 | <0.001 |
| DLCO%pred^g^ < 70 % | 1702/2434 (69.9) | 1093/1518  (72) | 609/916  (66.5) | 2434 | 0.004 |
| 6-minute walking distance, meters | 430.9 (134.7) | 418.9 (134.8) | 449.3 (132.5) | 975 | <0.001 |
| Other disease characteristics | | | | | |
| CRP^h^ elevation  > 5 mg/L | 630/3170  (19.9) | 415/1951  (21.3) | 215/  (17.6) | 3170 | 0.013 |
| Pulmonal hypertension on echocardiography (sPAP^i^ > 45 mmHg) | 212/2104  (10.1) | 142/1343  (10.6) | 70/761  (9.2) | 2104 | 0.314 |
| Heart dysfunction (LVEF^j^ < 50%) | 254/2677  (9.5) | 162/1689  (9.6) | 92/988  (9.3) | 2677 | 0.812 |
| Active esophageal symptoms at baseline^k^ | 2857/3194 (89.4) | 1875/1970 (95.2) | 982/1224 (80.2) | 3194 | <0.001 |
| Stomach symptoms^l^ | 857/3150  (27.2) | 571/1948  (29.3) | 286/1202  (23.8) | 3150 | <0.001 |
| Intestinal symptoms^l^ | 900/3162  (28.5) | 582/1949  (29.9) | 318/1213  (26.2) | 3162 | 0.027 |
| **Supplementary Table S1** Baseline characteristics of SSc-ILD GERD subpopulation with and without PPI  Variables are presented as n (percentages) or as means (SD). *p values were obtained using students t-test or Chi-square test. ^a^GERD, gastroesophageal reflux disease; ^b^SSc, systemic sclerosis; ^c^assessment of cutaneous form according LeRoy classification [2]; ^d^ILD (interstitial lung disease) modifying treatment includes cyclophosphamide, mycophenolate mofetil, tocilizumab, rituximab, nintedanib, autologous stem cell transplantation and lung transplantation [3]; ^e^NYHA, New York Heart Association [1]; ^f^FVC%pred, % predicted forced vital capacity; ^g^DLCO%pred, % predicted diffusing capacity of lungs for carbon monoxide; ^h^CRP, c-reactive protein; ^i^sPAP, systolic pulmonal arterial pressure; ^j^LVEF, left ventricular ejection fraction; ^k^active esophageal symptoms included patient reported reflux and/or dysphagia; ^l^stomach and intestinal symptoms were defined by past medical history. | | | | | |

|  | Total  n = 4400 | ≥ 1 follow-up over 12±3 months  n = 1691 | Follow-up but not over 12±3 months  n = 878 | no follow-up  n = 1008 | Excluded due to missing data at follow-up or PH^a^ in RHC^b^  n = 823 | N data available |
| --- | --- | --- | --- | --- | --- | --- |
| Female sex | 3688/4399  (83.8) | 1396/1691  (82.6) | 749/878  (85.3) | 830/1007  (82.4) | 713/823 (86.6) | 4399 |
| Age at baseline, years | 57.3  (13.2) | 55.3  (12.9) | 57.2 (13.2) | 59.4  (13.3) | 58.9 (13.1) | 4400 |
| Ever smoker | 1147/3276  (35.0) | 391/1010  (38.7) | 219/706  (31.0) | 325/966  (33.6) | 212/594 (35.7) | 3276 |
| Disease duration, years | 10.2  (8.8) | 9.5  (8.6) | 10.2  (8.5) | 10.7  (9.0) | 11.3 (9.3) | 3837 |
| Diffuse cutaneous SSc^cd^ | 1695/3378  (50.2) | 625/1225  (51.0) | 352/678  (51.9) | 417/836  (49.9) | 301/639 (47.1) | 3378 |
| Anti- Centromere^d^ | 798/3837  (20.8) | 285/1536  (18.6) | 143/763  (18.7) | 209/833  (25.1) | 161/705 (22.8) | 3837 |
| Anti-Topoisomerase 1^d^ | 2043/3936 (51.9) | 843/1558  (54.1) | 429/791  (54.2) | 442/879  (50.3) | 329/708 (46.5) | 3936 |
| Treatment | | | | | | |
| ILD modifying treatment^e^ | 1308/3230 (40.5) | 361/975  (37.0) | 297/709  (41.9) | 432/956  (45.2) | 218/590 (36.9) | 3230 |
| Corticosteroids | 1216/3230 (37.6) | 333/975  (34.2) | 298/709  (42.0) | 372/956  (38.9) | 213/590 (36.1) | 3230 |
| Proton pump inhibitors | 1987/3230  (61.5) | 577/975  (59.2) | 451/709  (63.6) | 587/956  (61.4) | 372/590 (63.1) | 3230 |
| Lung characteristics | | | | | | |
| Dyspnea NYHA^f^ > 2 | 616/3956  (15.6) | 162/1572  (10.3) | 131/798  (16.4) | 157/853  (18.4) | 166/733 (22.6) | 3956 |
| FVC%pred^g^ at baseline | 85.8  (22.1) | 88.0 (21.4) | 84.7  (22.0) | 85.2  (22.5) | 82.0 (22.6) | 3729 |
| DLCO%pred^h^ at baseline | 60.8  (19.7) | 62.9 (18.5) | 61.1 (20.5) | 60.5  (20.1) | 55.0 (20.5) | 3447 |
| Other disease characteristics | | | | | | |
| CRP^i^ elevation > 5 mg/L | 897/4321  (20.8) | 338/1657  (20.4) | 179/862  (20.8) | 192/999  (19.2) | 188/803 (23.4) | 4321 |
| Active esophageal symptoms at baseline^j^ | 3834/4352 (88.1) | 1454/1679 (86.6) | 782/869 (90.0) | 873/993 (87.9) | 725/811 (89.4) | 4352 |
| Stomach symptoms^k^ | 1174/4299  (27.3) | 445/1660  (26.8) | 247/861  (28.7) | 261/974  (26.8) | 221/804 (27.5) | 4299 |
| Intestinal symptoms^k^ | 1202/4312  (27.9) | 428/1668  (25.7) | 274/864  (31.7) | 279/975  (28.6) | 221/805 (27.5) | 4312 |
| Death | 416/3392  (12.3) | 192/1691  (11.4) | 89/878  (10.1) | - | 135/823 (16.4) | 3392 |
| **Supplementary Table S2** Comparison of SSc-ILD GERD patients with and without follow-up  Variables are presented as n (percentages) or as means (SD). ^a^PH, pulmonary hypertension; ^b^RHC right heart catherization; ^c^SSc, systemic sclerosis; ^d^assessment of cutaneous form according LeRoy classification [2]; ^e^ILD (interstitial lung disease) modifying treatment includes cyclophosphamide, mycophenolate mofetil, tocilizumab, rituximab, nintedanib, autologous stem cell transplantation and lung transplantation [3]; ^f^NYHA, New York Heart Association [1]; ^g^FVC%pred, % predicted forced vital capacity; ^h^DLCO%pred, % predicted diffusing capacity of lungs for carbon monoxide; ^i^CRP, c-reactive protein; ^j^active esophageal symptoms included patient reported reflux and/or dysphagia; ^k^stomach and intestinal symptoms were defined by past medical history. | | | | | | |

|  | Female | Male | Total | N data available | p value* |
| --- | --- | --- | --- | --- | --- |
| Δ FVC1-FVC2^a^,  mean (SD) | 3.8 (11.7) | 2.9 (10.5) | 3.6 (11.5) | 1711 | 0.208 |
| FVC%pred^b^ relative decline  ≥ 10%, n(%) | **451 (31.9)** | **74 (25)** | **525 (30.7)** | **1711** | **0.020** |
| Δ DLCO1-DLCO2^a^,  mean (SD) | 2.1 (12.2) | 2.2 (9.7) | 2.1 (11.7) | 1471 | 0.859 |
| DLCO%pred^c^ relative decline  ≥ 15%, n(%) | 252 (21) | 58 (21.5) | 310 (21.1) | 1471 | 0.856 |
| **Supplementary Table S3** Changes in FVC and DLCO according to sex in longitudinal SSc-ILD subpopulation with GERD *p values obtained using students t-test. ^a^FVC1 and DLCO1 respectively were collected at start of 12±3 month interval; FVC2 and DLCO2 were collected at end of 12±3 month interval; 12±3 month intervals in longitudinal population for Goh Criteria were taken; ^b^FVC%pred, %predicted forced vital capacity; ^c^DLCO%pred, %predicted diffusing capacity of lungs for carbon monoxide | | | | | |

|  | Progression of ILD^a^ over 12±3 months  n = 531 | |
| --- | --- | --- |
| Covariates | HR* [95% CI] | p value* |
| Female sex | **1.34 [1.03-1.76]** | **0.030** |
| Age at baseline, years | **1.02 [1.01-1.03]** | **<0.001** |
| Disease duration, years | 0.99 [0.98-1.00] | 0.054 |
| Diffuse cutaneous SSc^bc^ | 1.09 [0.86-1.37] | 0.464 |
| Anti-Topoisomerase 1^c^ | 0.97 [0.80-1.19] | 0.790 |
| Ever smoker | 0.96 [0.76-1.21] | 0.744 |
| Dyspnea NYHA^d^ > 2 | 1.14 [0.86-1.51] | 0.364 |
| FVC%pred^e^ at baseline | **0.99 [0.99-1.00]** | **0.048** |
| DLCO%pred^f^ at baseline | 1.00 [0.99-1.00] | 0.590 |
| ILD modifying treatment^g^ | 1.08 [0.87-1.33] | 0.485 |
| Proton pump inhibitors | 0.98 [0.73-1.32] | 0.885 |
| **Supplementary Table S4** Predictive factors for progression of ILD in SSc-ILD patients with GERD and active gastroesophageal symptoms at baseline  *p values and HR (hazard ratios) were obtained using cox regression analyses with multiple imputations. ^a^ILD, interstitial lung disease; ^b^SSc, systemic sclerosis; ^c^assessment of cutaneous subset according LeRoy classification [23]; ^d^NYHA, New York Heart Association [25]; ^e^FVC%pred, % predicted forced vital capacity; ^f^DLCO%pred, % predicted diffusing capacity of lungs for carbon monoxide; ^g^ILD modifying treatment includes cyclophosphamide, mycophenolate mofetil, tocilizumab, rituximab, nintedanib, autologous stem cell transplantation and lung transplantation [26]. | | |

|  | Death (1)  n = 164 | | Progression of ILD^a^ over 12±3 months or death (2)  n = 615 | |
| --- | --- | --- | --- | --- |
| Covariates | HR* [95% CI] | p value* | HR* [95% CI] | p value* |
| Female sex | 0.72 [0.44-1.19] | 0.200 | **1.32 [1.03-1.69]** | **0.030** |
| Age at baseline, years | **1.07 [1.05-1.08]** | **<0.001** | **1.02 [1.01-1.03]** | **<0.001** |
| Disease duration, years | 1.01 [0.99-1.03] | 0.455 | 0.99 [0.98-1.00] | 0.103 |
| Diffuse cutaneous SSc^bc^ | 1.42 [0.90-2.24] | 0.133 | 1.12 [0.90-1.39] | 0.314 |
| Anti-Topoisomerase 1^c^ | 1.42 [0.97-2.08] | 0.073 | 0.97 [0.81-1.18] | 0.788 |
| Ever smoker | 1.21 [0.69-2.12] | 0.496 | 0.99 [0.79-1.23] | 0.900 |
| Dyspnea NYHA^d^ > 2 | 1.18 [0.74-1.87] | 0.489 | 1.14 [0.88-1.48] | 0.327 |
| FVC%pred^e^ at baseline | 0.99 [0.98-1.00] | 0.109 | **0.99 [0.99-1.00]** | **0.016** |
| DLCO%pred^f^ at baseline | **0.97 [0.96-0.98]** | **<0.001** | 1.00 [0.99-1.00] | 0.212 |
| ILD modifying treatment^g^ | 0.73 [0.44-1.23] | 0.235 | 1.04 [0.85-1.27] | 0.690 |
| Proton pump inhibitors | 1.13 [0.75-1.71] | 0.559 | 0.99 [0.76-1.29] | 0.922 |
| **Supplementary Table S5** Predictive factors for mortality (1) and progression of ILD or death (2) in SSc-ILD patients with GERD and active gastroesophageal symptoms at baseline  *p values and HR (hazard ratios) were obtained using cox regression analyses with multiple imputations. ^a^ILD, interstitial lung disease; ^b^SSc, systemic sclerosis; ^c^assessment of cutaneous form according LeRoy classification [2]; ^d^NYHA, New York Heart Association [1]; ^e^FVC%pred, % predicted forced vital capacity; ^f^DLCO%pred, % predicteddiffusing capacity of lungs for carbon monoxide; ^g^ILD modifying treatment includes cyclophosphamide, mycophenolate mofetil, tocilizumab, rituximab, nintedanib, autologous stem cell transplantation and lung transplantation [3]. | | | | |

|  | Progression of ILD^a^ over 12±3 months  n = 902 | |
| --- | --- | --- |
| Covariates | HR [95% CI] | p value |
| Female sex | **1.22 [1.00-1.49]** | **0.046** |
| Age at baseline, years | **1.02 [1.01-1.02]** | **<0.001** |
| Disease duration, years | **0.99 [0.98-1.00]** | **0.016** |
| Diffuse cutaneous SSc (LeRoy) | 1.05 [0.88-1.24] | 0.596 |
| Anti-topoisomerase 1 antibodies | 0.99 [0.85-1.16] | 0.911 |
| Ever smoker | 0.99 [0.80-1.22] | 0.909 |
| Dyspnea NYHA > 2 | 1.22 [0.99-1.51] | 0.062 |
| FVC at baseline | **0.99 [0.99-1.00]** | **0.006** |
| DLCO at baseline | 1.00 [0.99-1.00] | 0.423 |
| ILD modifying drugs | 1.02 [0.86-1.20] | 0.837 |
| Proton pump inhibitors | 0.99 [0.85-1.16] | 0.937 |
| **Supplementary Table S6** Predictive factors for progression of ILD in whole SSc-ILD cohort (including GERD with PPI, GERD without PPI, nonGERD with PPI, nonGERD without PPI). *p values and HR (hazard ratios) were obtained using cox regression analyses with multiple imputations. ^a^ILD, interstitial lung disease; ^b^SSc, systemic sclerosis; ^c^assessment of cutaneous subset according LeRoy classification [23]; ^d^NYHA, New York Heart Association [25]; ^e^FVC%pred, % predicted forced vital capacity; ^f^DLCO%pred, % predicted diffusing capacity of lungs for carbon monoxide; ^g^ILD modifying treatment includes cyclophosphamide, mycophenolate mofetil, tocilizumab, rituximab, nintedanib, autologous stem cell transplantation and lung transplantation [26]. | | |

|  | Progression of ILD (Goh Criteria) over 12±3 months  n = 160 | |
| --- | --- | --- |
| Covariates | HR [95% CI] | p value |
| Female sex | 1.11 [0.71-1.73] | 0.640 |
| Age at baseline, years | **1.03 [1.01-1.04]** | **<0.001** |
| Disease duration, years | 0.98 [0.95-1.01] | 0.167 |
| Diffuse cutaneous SSc (LeRoy) | 0.95 [0.64-1.41] | 0.806 |
| Anti-topoisomerase 1 antibodies | 0.96 [0.68-1.34] | 0.795 |
| Ever smoker | 0.97 [0.62-1.52] | 0.905 |
| Dyspnea NYHA > 2 | 1.49 [0.80-2.78] | 0.210 |
| FVC at baseline | 0.99 [0.98-1.00] | 0.130 |
| DLCO at baseline | 1.00 [0.99-1.01] | 0.431 |
| ILD modifying treatment | 0.80 [0.54-1.19] | 0.274 |
| Proton pump inhibitors | 0.77 [0.48-1.25] | 0.289 |
| **Supplementary Table S7** Predictive factors for progression of ILD in non GERD SSc-ILD patients (nonGERD without PPI). *p values and HR (hazard ratios) were obtained using cox regression analyses with multiple imputations. ^a^ILD, interstitial lung disease; ^b^SSc, systemic sclerosis; ^c^assessment of cutaneous subset according LeRoy classification [23]; ^d^NYHA, New York Heart Association [25]; ^e^FVC%pred, % predicted forced vital capacity; ^f^DLCO%pred, % predicted diffusing capacity of lungs for carbon monoxide; ^g^ILD modifying treatment includes cyclophosphamide, mycophenolate mofetil, tocilizumab, rituximab, nintedanib, autologous stem cell transplantation and lung transplantation [26]. | | |

Supplementary Figure S1: Progression-free survival curve in SSc-ILD subpopulation with and without GERD. N at risk are represented below.

Supplementary Figure S2: Overall survival of SSc-ILD subpopulation with GERD according to PPI usage and follow-up at any time. N at risk are represented below.

**REFERENCES**

1. New York Heart A. Diseases of the heart and blood vessels: nomenclature and criteria for diagnosis. Boston, 1964.

2. LeRoy EC, Black C, Fleischmajer R, Jablonska S, Krieg T, Medsger TA, et al. Scleroderma (systemic sclerosis): classification, subsets and pathogenesis. J Rheumatol. 1988 Feb; 15(2):202-205.

3. Roofeh D, Lescoat A, Khanna D. Treatment for systemic sclerosis-associated interstitial lung disease. Curr Opin Rheumatol. 2021 May 01; 33(3):240-248.

4. Maron BA. Revised Definition of Pulmonary Hypertension and Approach to Management: A Clinical Primer. J Am Heart Assoc. 2023 Apr 18; 12(8):e029024.

List of EUSTAR Collaborators

| Serena Guiducci | Florence (Italy) |
| --- | --- |
| Ulrich Walker | Basel (Switzerland) |
| Florenzo Iannone | Bari (Italy) |
| Radim Becvar | Prague (Czech) |
| Otylia Kowal Bielecka | Bialystok (Poland) |
| Maurizio Cutolo | Genova (Italy) |
| Francesco Ciccia | Naples (Italy) |
| Elise Siegert | Berlin (Germany) |
| Simona Rednic | Cluj-Napoca (Romania) |
| Yannick Allanore | Paris (France) |
| Panayiotis Vlachoyiannopoulos | Athens (Greece) |
| Carlomaurizio Montecucco | Pavia (Italy) |
| Murat Inanc | Istanbul (Turkey) |
| Maria Martin, Beatriz Joven, Cioly Mendez | Madrid (Spain) |
| Srdan Novak | Rijeka (Croatia) |
| Gábor Kumánovics | Pecs (Hungary) |
| Michele Iudici | Geneva (Switzerland) |
| Przemyslaw Kotyla | Katowice (Poland) |
| Elisabetta Zanatta | Padova (Italy) |
| Katja Perdan-Pirkmajer | Ljublijana (Slovenia) |
| Bernard Coleiro | Balzan (Malta) |
| Silvia Svegliati, Devis Benfaremo, Chiara Paolini, Silvia Agarbati | Ancona (Italy) |
| Dominique Farge Bancel | Paris (France) |
| Paolo Airò | Brescia (Italy) |
| Kristofer Andréasson | Lund (Sweden) |
| Mislav Radic | Split (Croatia) |
| Alexandra Balbir-Gurman | Haifa (Israel) |
| Andrea Lo Monaco | Ferrara (Italy) |
| Nicolas Hunzelmann | Köln (Germany) |
| Annamaria Iagnocco | Torino (Italy) |
| Luca Idolazzi | Verona (Italy) |
| Josko Mitrovic | Zagreb (Croatia) |
| Voon Ong, Annalyn Nunag | London (United) |
| Hanneke Knaapen, Sander van Leuven, Rogier Thurlings | Nijmegen (The) |
| Jelena Colic | Belgrade (Serbia) |
| Jörg Henes | Tübingen (Germany) |
| Vera Ortiz-Santamaria | Barcelona (Spain) |
| Johannes Pflugfelder | Stuttgart (Germany) |
| Dorota Krasowska | Lublin (Poland) |
| Samuel Rubeli | Aarau (Switzerland) |
| Michaela Köhm | Frankfurt (Germany) |
| Ivan Foeldvari | Hamburg (Germany) |
| Gianluigi Bajocchi | Reggio (Italy) |
| José António Pereira da Silva | Coimbra (Portugal) |
| Bojana Stamenkovic | Niska (Serbia) |
| Antonio Tonutti, Francesca Motta | Rozzano (Italy) |
| Claudia Ickinger, Nimmisha Govind | Johannesburg (South) |
| Lidia P. Ananieva | Moscow (Russia) |
| Michael Hughes | Salford (United) |
| Philipp Klemm, Ulf Müller-Ladner | Bad (Germany) |
| Klaus Søndergaard | Aarhus (Denmark) |
| - | Genova (Italy) |
| Merete Engelhart | Hellerup (Denmark) |
| Gabriella Szücs | Debrecen (Hungary) |
| Carlos de la Puente | Madrid (Spain) |
| Øyvind Midtvedt, Torhild Garen, Håvard Fretheim, Mona-Lovise Talaro Ramsli | Oslo (Norway) |
| David Launay | Lille (France) |
| Valeria Riccieri | Rome (Italy) |
| Andra Balanescu | Bucharest (Romania) |
| Ami A. Shah | Baltimore (USA) |
| Ana Maria Gheorghiu | Bucharest (Romania) |
| Andreas Wirsching, Janina Auth, Alina Ramming, Havvanur Kartalcik | Erlangen (Germany) |
| Francesca Ingegnoli | Milan (Italy) |
| Bertrand Dunogue, Benjamin Chaigne | Paris (France) |
| Vanessa Smith | Gent (Belgium) |
| Francesco Paolo Cantatore | Foggia (Italy) |
| Mette Mogensen | Copenhagen (Denmark) |
| Carlos Alberto von Mühlen | Porto (Brazil) |
| Felix Lauffer | Munich (Germany) |
| Piotr Wiland | Wroclaw (Poland) |
| Marie Vanthuyne | Brussels (Belgium) |
| Juan Jose Alegre-Sancho | Valencia (Spain) |
| Martin Aringer | Dresden (Germany) |
| Ellen De Langhe | Leuven (Belgium) |
| Branimir Ani? | Zagreb (Croatia) |
| Sule Yavuz | Altunizade-Istanbul (Turkey) |
| Brigitte Granel | Marseille (France) |
| Carolina de Souza Müller | Curitiba (Brazil) |
| Svetlana Agachi | Chisinau (Republic) |
| Margarita Pileckyte | Kaunas (Lithuania) |
| Simon Stebbings | Dunedin (New) |
| Alessandra Vacca | Monserrato (Italy) |
| Percival D. Sampaio-Barros | São (Brazil) |
| Kamal Solanki | Hamilton (New) |
| Douglas Veale | Dublin (Ireland) |
| Esthela Loyo | Santiago (Dominican) |
| Walid Ahmed Abdel Atty Mohamed | Alexandria (Egypt) |
| Jacek Olas | Cracow (Poland) |
| Edoardo Rosato | Rome (Italy) |
| Figen Yargucu Zhini | Bornova (Turkey) |
| Cristina-Mihaela Tanaseanu | Bucharest (Romania) |
| Rosario Foti | Catania (Italy) |
| Codrina Ancuta | Iasi (Romania) |
| Britta Maurer | Bern (Switzerland) |
| Marzena Olesinska | Warsaw (Poland) |
| Cristiane Kayser | São (Brazil) |
| Nihal Fathi | Assiut (Eqypt) |
| Jorge Juan González Martín | Madrid (Spain) |
| Sophie Blaise | Grenoble (France) |
| Patricia Senet | Paris (France) |
| Emmanuel Chatelus | Strasbourg (France) |
| Ira Litinsky | Tel-Aviv (Israel) |
| Martial Koenig, Sabrina Hoa, Jean-Luc Senécal | Montreal (Canada) |
| Rajvinder Cheema, Begonya Alcacher Pitarch, Lorraine Green, Vishal Kakkar, Stefano Di Donato | Leeds (United) |
| Goda Seskute | Vilnius (Lithuania) |
| Lesley Ann Saketkoo | New (USA) |
| Eduardo Kerzberg | Buenos (Argentina) |
| Breno Valdetaro Bianchi | Rio (Brazil) |
| Ivan Castellví | Barcelona (Spain) |
| Jasminka Milas-Ahic, Roberta Visevic | Osijek (Croatia) |
| Massimiliano Limonta | Bergamo (Italy) |
| Doron Rimar | Haifa (Israel) |
| Maura Couto | Viseu (Portugal) |
| Camillo Ribi | Lausanne (Switzerland) |
| Antonella Marcoccia | Roma (Italy) |
| Sarah Kahl | Bad (Germany) |
| Vivien M. Hsu | New (USA) |
| Thierry Martin | Strasbourg (France) |
| Sergey Moiseev | Moscow (Russia) |
| Lorinda S Chung | Stanford (USA) |
| Tim Schmeiser | Wuppertal-Elberfeld (Germany) |
| Dominik Majewski | Poznan (Poland) |
| Anna Wojteczek | Gdansk (Poland) |
| Julia Martínez-Barrio | Madrid (Spain) |
| Dinesh Khanna | Ann (USA) |
| Ana Catarina Rodrigues | Lisbon (Portugal) |
| Gabriela Riemekasten | Lübeck (Germany) |
| Lelita Santos | Coimbra (Portugal) |
| Yair Levy | kfar-saba (Israel) |
| Elena Rezus | Ia?i (Romania) |
| Daniel Brito De Araujo | Pelotas (Brazil) |
| Rossella Talotta, Sara Bongiovanni | Milan (Italy) |
| Marek Brzosko | Szczecin (Poland) |
| Prof. Hadi Poormoghim | Tehran (Iran) |
| Marta Mamani | Buenos (Argentina) |
| Ina Kötter | Hamburg (Germany) |
| Giovanna Cuomo | Napoli (Italy) |
| Oscar Massimiliano Epis | Milan (Italy) |
| Petros Sfikakis | Athens (Greece) |
| Juliana Markus | Uberlandia (Brazil) |
| Daniel Furst | Los (USA) |
| Ana-Maria Ramazan | Constanta (Romania) |
| Hans Ulrich Scherer, Tom WJ Huizinga | Leiden (The) |
| Estibaliz Lazaro | Bordeaux (France) |
| Alain Lescoat | Rennes (France) |
| Marco Matucci-Cerinic | Milano (Italy) |
| Julia Spierings | Ultrecht (The) |
| Lidia Rudnicka | Warsaw (Poland) |
| Susana Oliveira | Amadora (Portugal) |
| Fabiola Atzeni | Messina (Italy) |
| Masataka Kuwana | Tokyo (Japan) |
| Arsene Mekinian | Paris (France) |
| Mickaël Martin | Poitiers (France) |
| Yoshiya Tanaka | Kitakyushu (Japan) |
| Hidekata Yasuoka | Aichi (Japan) |
| Carmen-Pilar Simeón Aznar | Barcelona (Spain) |
| Tatsuya Atsumi | Sapporo (Japan) |
| Magda Pârvu | Bucharest (Romania) |
| Gonçalo Boleto | Lisbon (Portugal) |
| Nicoletta Del Papa | Milan (Italy) |
| Kastriot Kastrati | Vienna (Austria) |
| Jennifer Ben Shimol | Tel (Israel) |
| Anna Bazela-Ostromecka | Grunwaldzka (Poland) |
| Enrico Selvi | Siena (Italy) |
| Yasushi Kawaguchi | Tokyo (Japan) |
| Tomas Soukup | Hradec (Czech) |
| Andrea Nuñez Conde | Barcelona (Spain) |
| Marija Geroldinger-Simic | Linz (Austria) |
| Ignasi Rodríguez-Pintó | Barcelona (Spain) |
| Karen Voigt | Hamburg (Germany) |
| Torsten Kubacki | Köln (Germany) |
| Olena Garmish | Kiev (Ukraine) |
| Marta Mosca | Pisa (Italy) |
| Ulrich Gerth | Rheinfelden (Switzerland) |
| Marta Dzhus | Kiev (Ukraine) |
| Tomonori Ishii | Sendai (Japan) |
| Duygu Temiz Karadag | Kocaeli (Turkey) |
| Anastas Batalov | Plovdiv (Bulgaria) |
| Knarik Ginosyan | Yerevan (Armenia) |
| Vahan Mukuchyan | Yerevan (Armenia) |
| Valentina Vardanyan | Yerevan (Armenia) |
| Armine Haroyan | Yerevan (Armenia) |
| Tuulikki Sokka-Isler | Jyväskylä (Finland) |
| Len Harty | Cork (Ireland) |
| Mariela Geneva-Popova | Plovdiv (Bulgaria) |
| Mohammad Naffaa | Naharyia (Israel) |
| Cristina Maglio | Gothenburg (Sweden) |
| Cristiana Isabel Sieiro Santos | León (Spain) |
| Okada Masato, Futoshi Iwata | Tokyo (Japan) |
| Monique Hinchcliff | North (USA) |
| Samar Tharwat | Dakahlia (Egypt) |
| Ana Cordeiro | Almada (Portugal) |
| Roberto Giacomelli | Rome (Italy) |
| Francesco Benvenuti | Vicenza (Italy) |
